# Treatment of animal models of Alzheimer’s disease with extracellular vesicles or exosomes and involvement of microRNAs

**DOI:** 10.4103/NRR.NRR-D-25-01116

**Published:** 2026-01-27

**Authors:** Bridget Martinez, Philip V. Peplow

**Affiliations:** 1 Department of Pharmacology, University of Nevada-Reno, Reno, NV, USA; 2 Department of Medicine, University of Nevada-Reno, Reno, NV, USA; 3 Department of Anatomy, University of Otago, Dunedin, New Zealand

**Keywords:** Alzheimer’s disease, animal models, exosomes, extracellular vesicles, microRNAs, treatment outcomes

## Abstract

Alzheimer’s disease is a complex and devastating neurodegenerative disorder that accounts for roughly 80% of all dementia cases. It is primarily marked by the accumulation of senile amyloid-β plaques and neurofibrillary tangles composed of hyperphosphorylated tau protein. These pathological features are accompanied by chronic neuroinflammation and glial cell dysfunction, which collectively contribute to the progressive loss of synapses and neurons. As a result, individuals with Alzheimer’s disease experience gradual memory loss and cognitive decline. Currently, the global patient population is nearing 50 million, a number expected to increase dramatically over the coming decades. Conventional treatments focus on symptom management through acetylcholinesterase inhibitors, such as donepezil, galantamine, and rivastigmine, and the N-methyl-D-aspartate receptor antagonist memantine. However, the past few years have seen the approval of newer agents such as sodium oligomannate, aducanumab, and lecanemab, which show some promise in slowing disease progression. Unfortunately, most patients are not diagnosed until moderate or advanced stages when irreversible brain damage has occurred. This highlights an urgent need for early diagnosis and biomarkers together with therapeutic strategies aimed at early-stage intervention and identifying novel drug targets that address prodromal and established forms of the disease. This article is a literature review of extracellular vesicles/exosomes treatment in animal models of Alzheimer’s disease involving microRNAs. In the *in vivo* animal studies of Alzheimer’s disease reviewed, extracellular vesicles and exosomes from various sources improved memory and cognitive decline, lowered inflammation and amyloid deposition, and increased neuron survival in the brain. Loading extracellular vesicles and exosomes with microRNA mimics (e.g., miR-22, -29b, -124, -132, -138-5p, -342-5p, -711, and -7670-3p) or antagomirs (e.g., miR-206-antagomir) improved outcomes in animal models of Alzheimer’s disease. Supporting results were found in the *in vitro* cell studies reviewed.

## Introduction

Alzheimer’s disease (AD) is the most common neurodegenerative disorder, accounting for approximately 80% of dementia cases. It is characterized by the deposition in the brain of senile amyloid-β (Aβ) plaques, neurofibrillary tangles containing highly phosphorylated tau, chronic inflammation, glial dysfunction, along with associated loss of synapses and neurons, resulting in memory loss and cognitive dysfunction (Guo et al., 2020b; Jorfi et al., 2023). Braak and Thal staging systems classify the progression of neurological changes in AD based on the spread of neurofibrillary tangles (Braak et al., 2006) and Aβ plaques (Thal et al., 2002) throughout the brain. The number of AD patients worldwide is about 50 million (Wang et al., 2023). Early AD therapy uses acetyl cholinesterase inhibitors (donepezil, galantamine, and rivastigmine) and N-methyl-D-aspartate receptor antagonist (memantine) (Scheltens et al., 2021; Stoiljkovic et al., 2021). Recently, three approved AD therapeutic drugs (sodium oligomannate, aducanumab, and lecanemab) have been shown to slow AD progression (Syed, 2020; Valiukas et al., 2022; van Dyck et al., 2023). Unfortunately, most patients are not diagnosed until the disease has caused the brain to be irreversibly damaged (Guest et al., 2020). New biomarkers and potential therapies for prodromal and established AD are needed (Song et al., 2022).

Mesenchymal stem cells (MSCs) have anti-inflammatory and neuroprotection potential in the treatment of AD (Salwa and Kumar, 2021). However, MSC-based therapy is restricted by heterogeneity of the cells, immune response and low survival of transplanted cells, and ethical issues (Eliopoulos et al., 2005; Levy et al., 2020; Pajer et al., 2020). Extracellular vesicles (EVs) are nanometer-sized cell-secreted vesicles and play a critical role in neurodegenerative diseases (Izadpanah et al., 2018; Vinaiphat and Sze, 2019; Bahmani and Ullah, 2022). EVs are membrane-bound vesicles that transport their cargoes for intercellular communication such as proteins, messenger RNAs (mRNAs), microRNAs (miRNAs), and phospholipids that are reflective of their producer cells (Qiu et al., 2019).

EVs are secreted by nearly all cell types and among them, MSCs-derived EVs (MSC-EVs) are easily obtained and maintained (Koniusz et al., 2016; Szatanek et al., 2017). MSC-EVs have more advantages in therapeutic potential than MSCs, including low immunogenicity, high stability, long half-life in the circulation, low tumorigenicity, and easy passage through the blood–brain barrier (BBB) (Ding et al., 2023). MSC-EVs ameliorate cognitive impairments in AD mice by reducing Aβ accumulation (Cone et al., 2021), relieving neuronal damage (Ma et al., 2020), and decreasing inflammation and oxidative stress (Bodart-Santos et al., 2019; Losurdo et al., 2020). MSC-EVs avoid the side effects associated with stem cell transplantation. Although MSC-EVs represent an ideal potential therapy for AD (Cone et al., 2021), nonuniform treatment outcomes and low efficiency have been found (Yin et al., 2023). The therapeutic efficacy of MSC-EVs can be improved by loading therapeutic molecules such as nucleic acids, peptides and proteins into native EVs.

Astrocytes constitute a large proportion of the cell population in the central nervous system. Aβ oligomers induce astrocyte overactivation, affect intracellular calcium homeostasis, impair mitochondrial function, increase the production of reactive oxygen and nitrogen species, as well as the release of toxic glutamine and proinflammatory factors (Phatnani and Maniatis, 2015; Guo et al., 2020b). A series of events in the reactive astrocytes eventually leads to synapse loss and neuron death. EVs are a major mediator in astrocyte-neuron communication. The EVs secreted by astrocytes contain many active molecules, such as proteins, phospholipids, mRNAs, miRNAs, and long-non-coding RNAs (lncRNAs), which play an important regulatory role in the physiological and pathological processes of the central nervous system. Astrocyte-derived EVs activated by interleukin-1β and tumor necrosis factor-α inhibit neurite outgrowth in the AD brain (Upadhya et al., 2020; You et al., 2020). Toxic proteins such as Aβ, apolipoprotein E epsilon 4 (ApoEε4), ceramide, and prostate apoptosis response 4 are released from astrocytes in EVs, and which spread in the whole brain to stimulate the aggregation of Aβ peptides and cause the death of neighboring neurons and glial apoptosis (Wang et al., 2012; Söllvander et al., 2016; Elsherbini et al., 2020; Upadhya et al., 2020).

Microglia are resident macrophages of the brain, and they continuously survey the brain for pathogens. Activation of microglial toll-like receptors by lipopolysaccharides or Aβ oligomers leads to the release of proinflammatory cytokines and reactive oxygen species (Wang et al., 2015), which drives the pathological progression of AD (Salminen et al., 2008, 2009; Heurtaux et al., 2010; Forloni and Balducci, 2018). Proinflammatory microglia (M1 phenotype) promote neuronal damage by producing reactive oxygen species, while anti-inflammatory microglia (M2 phenotype) promote neuronal survival by accelerating wound healing and tissue remodeling (Kobayashi et al., 2013; Plastira et al., 2016). MSC-EVs can prevent microglia activation through modulation of inflammatory cytokines (Jaimes et al., 2017).

EVs include exosomes, microvesicles, and apoptotic bodies, which are of different sizes (Garcia-Contreras and Thakor, 2023) and known to participate in intercellular communication (Paschon et al., 2016). An illustration showing the variety of EVs is included in two recent review articles (Du et al., 2023; Kumar et al., 2024). Exosomes are approximately 30-150 nm in size with a lipid bilayer membrane structure and released upon the fusion of a multivesicular body with the plasma membrane. Microvesicles have a diameter size of 150 nm–1 µm and apoptotic bodies are 1–5 µm in size. Exosomes can constitute a large part of the smaller EV fraction, often around 70%–80%. EVs are often characterized by the presence of specific surface markers such as CD9, CD63, and CD81 (Ekstrom et al., 2022). Distant intercellular communication occurs through RNA signals triggered by pathogenic materials of AD during development and progression (Zhou et al., 2018; Guo et al., 2020a). Exosomes transport mRNA, miRNA, and transcription factors to neighbouring cells and influence their cellular function (Yan et al., 2019) and exosome-derived miRNAs and lncRNAs are involved in the pathophysiology of neurodegenerative diseases (Beeraka et al., 2020). Exosomes establish communication between cells and exchange genetic materials (Wang et al., 2013) such as mRNAs, miRNAs, transfer RNAs and lncRNAs (Kumar et al., 2020) to modulate physiological processes such as immune response, inflammatory response, angiogenesis, apoptosis, blood coagulation, and cell debris removal (He et al., 2021). Exosomes possess low immunogenicity, high biocompatibility, and flexible targeting, making them ideal delivery vehicles and nanocarriers for oligonucleotide drugs such as miRNAs. They can bypass biological barriers and accumulate in pathological sites and are easily excreted from the body. They could have important roles in the diagnosis, prevention, and treatment of diseases (Elsharkasy et al., 2020). Due to exosomes being able to pass through the blood–brain barrier via transcytosis and other mechanisms, exosomes originating in the brain have been detected in the peripheral circulation, and their contents, such as miRNAs, could serve as potential diagnostic biomarkers of neurodegenerative diseases and psychiatric conditions (Abdelsalam et al., 2023).

MSC-EVs exert effects on brain diseases via the transfer of their carried miRNAs (Qiu et al., 2018). MiRNAs are single-stranded, short (20–22 nt), non-coding RNA molecules that regulate gene expression of their complementary mRNA targets by binding to the 3′-untranslated region (Bartel, 2004; Mattick and Makunin, 2006). MiRNAs play important roles in multiple biological processes, including cell cycle control, cell growth and differentiation, apoptosis, embryo development (Ambros, 2001; Brennecke et al., 2003; Bartel, 2004) and brain development (Bartel, 2009). MiRNAs are involved in AD pathogenesis, having strong potential of being therapeutic biomarkers for AD (Iranifar et al., 2019). Evidence indicates that miRNAs in EVs contribute to many cellular and biological processes, such as neuronal cell growth and apoptosis, thus affecting different functional processes such as learning and memory (Luceri et al., 2017). MiR-223 delivery from MSC-derived exosomes to an AD cell model protected against nerve injury (Wei et al., 2020, 2024). Several pathways and molecules, including RNA binding proteins, have been described that impact exosomal miRNA loading (Janas et al., 2020).

We have performed a PubMed literature search for the treatment of AD using EVs and exosomes and how loading with specific miRNA mimics or miRNA inhibitors affects outcomes and alteration of miRNAs in the brain. Multiple miRNAs, including miR-455-3p, miR-193b-3p, and miR-31-5p, inhibited the expression of amyloid precursor protein (APP) *in vivo* and *in vitro* by targeting APP 3′-untranslated region (Liu et al., 2014; Kumar et al., 2019; Barros-Viegas et al., 2020; Kumar and Reddy, 2021). Loading of EVs or exosomes with one or several of these miRNAs could be a potential therapy for AD. In pathological conditions, APP is proteolyzed by β- and γ-secretase, producing peptides of 40 or 42 amino acids (Aβ_1–40_ or Aβ_1–42_) (Selkoe, 2001). Both peptides, especially Aβ_1–42_, are hydrophobic and tend to fold in a β-sheet structure, which recruits other extracellular proteins to form senile plaques (Masters et al., 2015).

## Search Strategy

### Search strategy for extracellular vesicles

We performed a PubMed search for EVs and microRNAs for treating AD using the search terms “extracellular vesicles,” “microRNA,” and “Alzheimer’s disease.” The total number of articles found was 164, and they were published from February 2018 to November 2024. Of these articles, 12 were selected for the review; those not selected were reviews, meta-analyses, retracted articles, not performed with human/animal cells or animal models, not on AD, or written in a non-English language.

### Search strategy for exosomes

We performed a PubMed search for exosomes and microRNAs for treating AD using the search terms “exosomes,” “microRNA,” and “Alzheimer’s disease.” The total number of articles found was 161 and were published from February 2018 to January 2025. Of these articles, 13 were selected for the review; those not selected were reviews, meta-analyses, retracted articles, not performed with human/animal cells or animal models, not on AD, or written in a non-English language. While use of the term “exosome” is discouraged as it is likely that a broad grouping of EVs is being studied and not exosomes specifically (Welsh et al., 2024), we have kept this term as it had been used in the titles of the original articles and provided a way of separating the articles found in the PubMed searches into two sets.

### Extracellular vesicles to treat models of Alzheimer’s disease

Various *in vivo* animal models of AD have been used, including mice treated with amyloid-β peptide, streptozotocin, and rats treated with amyloid-β peptide. Streptozotocin injected intracerebroventricularly at a low sub-diabetogenic dose in mice was employed to establish a sporadic AD model. APP/PS1dE9 transgenic mice, 5×FAD transgenic mice and 3×Tg-AD mice were used. APP/PS1dE9 transgenic mice, 5×FAD transgenic mice and 3×Tg-AD mice are models of AD caused by mutations in specific genes. APP/PS1dE9 transgenic mice have mutations in APP (Swedish mutation), and presenilin 1 (exon 9 deletion), leading to rapid accumulation of amyloid plaques in the brain. The 5×FAD mouse model develops amyloid but not tau pathology due to three mutations in APP and two mutations in presenilin 1. The 3×Tg-AD mouse model develops amyloid and tau pathology due to mutations in APP, presenilin 1, and tau. All of these are well-established model systems for the study of AD (Oblak et al., 2021; Javonillo et al., 2022; Soto et al., 2023; Xu et al., 2023). Mice subjected to a repetitive mild traumatic brain injury (Xu et al., 2021), which is a risk factor for developing AD, have also been studied. An *in vitro* animal cell model was the mouse microglia cell line BV-2 primed with lipopolysaccharides and stimulated with Aβ aggregates (Liu et al., 2020). HT22 mouse hippocampal neurons stimulated with FGF-2 had been used.

### Exosomes to treat models of Alzheimer’s disease

*In vivo* animal models of AD were rats treated with AlCl3 by intragastric intubation, rats treated with amyloid-β peptide, and APP/PS1dE9 transgenic mice, 3×Tg-AD mice and 5×FAD mice. A repetitive traumatic brain injury model was used. An *in vitro* cell model was human neuroblastoma SH-SY5Y cells treated with amyloid-β peptide (Krishtal et al., 2019). *APP* mRNA was ectopically expressed in N2a mouse cells, and HT22 mouse hippocampal neurons were treated with exosomes isolated from the hippocampi of APP mice.

The experimental design in each of the studies reviewed, together with the findings, are presented in **Tables 1** and **2**. The isolated EVs and exosomes were examined by scanning and/or transmission electron microscopy and found to be nanosized particles with a characteristic spherical or cup-shaped appearance. Some studies had identified surface markers of exosomes, which showed them to be the main particle type in the exosomal samples prepared. As was noted previously, EVs constitute exosomes, microvesicles and apoptotic bodies and in which exosomes may constitute a large part of the smaller EV fraction.

**Table 1:** Treatment of animal models of Alzheimer’s disease with EVs

Isolation of EVs	Animal model of Alzheimer’s disease	Treatment with EVs	Changes in learning and memory, Aβ burden, inflammation, oxidative stress, and neuronal survival in treated animal models	Reference
***In vivo studies***				
***Injection of Aβ_1–42_ oligomer into the brain***				
MSCs isolated from femurs of 4-wk-old Sprague–Dawley rats, and EVs obtained. MiR-206 antagomir was loaded on to MSC-EVs using electroporation. MSC-EVs and miR-206 antagomir were incubated without electroporation, which was used as a control along with electroporated MSC-EVs.	4 μL of Aβ_1–42_ oligomer (2 μg/μL, 4 μg/side, 8 μg/mouse) injected into the bilateral hippocampus of 10-wk-old male C57BL/6J mice.	After 3 wk of brain injection of Aβ_1–42_ oligomer, the AD mice were randomly divided into 6 groups: sham, Aβ, Aβ+MSC-EVs, Aβ+MSC-EVs-NC, Aβ+MSC-EVs-antagomir, Aβ+antagomir. Mice were intranasally administered with MSC-EVs, MSC-EVs-NC, MSC-EVs-antagomir, miR-206 antagomir and volume-matched PBS every 2 d for 6 wk. The nasal mucous membrane was permeabilized with 3 μL hyaluronidase (100 U/mouse) for 30 min. Subsequently, each nostril received a total volume of ~8 μL of MSC-EVs (5.22×10^8^), MSC-EVs-NC (MSC-EVs loaded with antagomir NC), MSC-EVs-antagomir (MSC-EVs loaded with ~10 pmol miR-206 antagomir), miR-206 antagomir (~10 pmol) or PBS for 3 min. The Morris water maze test was used to assess cognitive abilities of AD mice.	On treating AD mice intranasally with MSC-EVs, after 6 h MSC-EVs were detected in the prefrontal cortex and hippocampus, being mainly distributed in neurons, and taken up more by microglia than astrocytes. MSC-EVs-miR-206 antagomir ameliorated cognitive decline and reduced the Aβ plaques burden in the hippocampal DG region and prefrontal cortex. MSC-EVs-miR-206 antagomir had no noticeable impact on the brain tissue morphology. MSC-EVs-antagomir miR-206 significantly downregulated miR-206-3p levels in the hippocampus and prefrontal cortex of AD mice, followed by upregulation of BDNF expression.	Peng et al., 2024
Bone marrow MSCs (BM-MSCs) were isolated from femurs and tibias of Sprague–Dawley rats, 5–6 wk. The BM-MSCs were cultured, centrifuged, and EVs isolated from the supernatant by ultracentrifugation.	Aβ_1–42_ oligomer (5 μg/μL) injected into lateral ventricle of Sprague–Dawley rats, 6–7 wk, 200–220 g, female/male ratio 1/2.	24 Sprague–Dawley rats were randomly allocated into 4 groups (*n* = 6/group): normal control group (wild-type WT), model group (AD), model control group (AD-negative control [NC]), and model treatment group (AD-EVs). 5 d after Aβ_1–42_ injection, rats in the AD-EVs group were injected with 30 μg EVs in 100 μL PBS at the same position in the lateral ventricle; rats in the AD-NC group were injected with the same amount of BM-MSC conditioned medium after GW4869 treatment. The injection was performed once a mon at the same time for 2 mon. Behavioral tests were performed 3 wk after the last injection.	Treating AD rats with MSC-EVs ameliorated cognitive decline and reduced the Aβ burden in cerebral cortex and hippocampus. Levels of proinflammatory cytokines in cerebral cortex were decreased after EVs treatment. Protein and mRNA levels of Aβ decomposition factors NEP and IDE were significantly increased in EVs-treated AD rats.	Sha et al., 2021
***i.c.v. injection of STZ***				
Human iPSC-MSCs were induced and cultured (Gao et al., 2017). Anion-exchange chromatography was used to isolate and purify MSC-sEVs (small EVs). The mCherry-CD63 fusion gene was transduced into MSCs by lentiviral vectors to generate mCherry-MSCs. mCherry-MSCs were used to isolate MSC-sEVs.	Sporadic AD (sAD) model established by bilateral i.c.v. injections of STZ 3 mg/kg in C57BL/6J mice, 11-mon-old.	C57BL/6J mice, 10-mon-old, were randomly divided into sham, STZ+PBS, and STZ+MSC-sEVs groups (*n* = 10). After 1 wk of acclimatization, mice in the STZ+PBS and STZ+MSC-sEVs groups received bilateral i.c.v. injections of STZ 3 mg/kg to establish sporadic AD (sAD) model. Mice in the sham group received two i.c.v. injections of saline. At 2 mon later, mice in the sAD+PBS and sAD+MSC-sEVs were intracistenally injected with PBS and MSC-sEVs, respectively, once every 2 wk for a total of 3 injections. At 1 wk after the last injection of MSC-sEVs, the mice underwent the Morris water maze test.	Following intracisternal injection, MSC-sEVs were detected in both the cortex and hippocampus at 24 h later. MSC-sEVs improved spatial memory and cognition in sAD mice. MSC-sEVs reduced neuroinflammation and Aβ deposition by mitigating microgliosis and inhibiting the NLRP3 pathway. MSC-sEVs decreased neuronal apoptosis in sAD mice.	Lin et al. 2024a
***Transgenic animals***				
Astrocytes derived from neonatal rats were divided into 3 groups. For the Aβ_1–42_ injury group, astrocytes were incubated with DMEM-F12+2% exosome-free FBS, and 4 μM Aβ_1–42_ for 72 h. For the Aβ_1–42_ and haFGF_14–154_ co-treatment group, astrocytes were incubated with DMEM-F12+2% exosome-free FBS, 4 μM Aβ_1–42_ and 100 ng/mL haFGF_14–154_ (Aβ+H) for 72 h. The volume matched vehicle was added to the control group. AEVs (AEVs_ctrl_, AEVs_Aβ_, and AEVs_Aβ+H_) were collected from the supernatant of astrocytes subjected to the different treatments.	7-mon-old male APP/PS1dE9 transgenic mice.	AEVs were intranasally administered to 7-mon-old APP/PS1 mice every 2 d for 6 wk, followed by Y-maze and Morris water maze tests.	AEVs were distributed in the olfactory bulb and entorhinal cortex 6 h after the intranasal administration. AEVs_Aβ+H_ ameliorated cognitive behavior deficits, alleviated brain Aβ burden, and promoted synaptic plasticity in AD mice. The differentially expressed miRNAs in AEVs were measured by qPCR (*n* = 3). The level of change of miR-206-3p was the most significant among the differentially expressed miRNAs with the level being significantly lower in AEVs_Aβ+H_ compared to AEVs_Aβ_. The intracellular miR-206-3p level was increased approximately 4.34-fold in astrocytes damaged by Aβ but returned to normal level by aFGF treatment.	Peng et al., 2022
Primary neurons were isolated from the cortices of brains of embryos removed from pregnant E18 Sprague Dawley rats, 10–12 wk. After 14 d of culture *in vitro*, the primary neurons became mature, and sEVs were isolated from the supernatant of the primary neurons using ultracentrifugation.	APP/PS1 male mice, 20 wk	sEVs were labeled with the fluorescent dye DIR. DIR-labeled sEVs were injected i.v. through the tail vein of APP/PS1 mice at a dose of 1.0 × 10^10^ p/g. APP/PS1 mice were injected with glutamate treated sEVs (APP/PS1-glutamate sEVs group) or GABA treated sEVs (APP/PS1-GABA sEVs group) through the tail vein at the same time every other day for 40 d. APP/PS1 mice were injected with Ctrl sEVs as a vehicle (APP/PS1-Ctrl sEVs group). Spatial learning and memory were evaluated using the Morris water maze test 40 d post sEV administration. MiRNAs were loaded into sEVs by SBI’s high-efficiency sEVs-Fect siRNA/miRNA Transfection Kit. MiR-132-3p deficiency occurs in AD and promotes its pathology. The miR-132-3p mimics or antagomir-132-3p loaded into sEVs were injected i.v. into APP/PS1 mice. A total of 300 μg of agomir-132 or 700 μg of antagomir-132 were encapsulated in 1 mg of sEVs. 7 consecutive i.v. injections of agomir-132- or antagomir-132-loaded sEVs were administered to the mice at intervals of 1 d apart. Cy3-labeled agomir-132 was injected into APP/PS1 mice. The brain was removed after 24 h to confirm whether agomir-132 loaded sEVs could be effectively delivered to neurons in the brain.	The Morris water maze test showed that GABA treatment of sEVs rescued the spatial memory deficits in APP/PS1 mice while glutamate treatment of the sEVs deteriorated it. Cy3-labeled agomir-132 loaded sEVs (red) and NeuN^+^ neurons (green) overlapped in the brain slices. Injections i.v. of agomir-132- or antagomir-132-loaded sEVs to APP/PS1 mice showed that miR-132 had a protective effect on neuron viability.	Dou et al., 2021
Human ADSCs were cultured until 90% confluent, and then subjected to complete media supplemented with 10% EVs-depleted FBS for another 72 h. The EVs were isolated by differential centrifugation.	APP/PS1 mice, female, 9 mon, 32–40 g, and C57BL/6 mice, male, 6 wk, ~20 g.	APP/PS1 mice were intranasally administered 10 μL ADSC-EVs at a protein dose of 1 mg/kg every 2 d for 2 wk. The EVs solution was given through a tube to the openings of the left and right nostrils sequentially drop by drop (0.5 μL/drop). The interval of administration was 1 min, and total dosing time lasted about 10 min to allow the mice to inhale all the preparations. APP/PS1 and wild type littermates given the same volume of PBS (the vehicle) every 2 d for 2 wk served as the negative and normal control, respectively.	^125^I-labeled EVs were found in all the detected brain areas, reaching a peak at 1 h after administration. In the olfactory bulb and anterior olfactory nucleus, EVs were mostly distributed in the neurons. A small number of EVs was found in microglia and astrocytes. In the cortex, EVs were accumulated in neurons and less in microglia and astrocytes. EVs efficiently rescued the memory and spatial learning deficits in AD mice. EVs ameliorated neurologic damage in the brain and increased neurogenesis in AD mice. EVs slightly reduced Aβ burden and decreased microglia activation.	Ma et al., 2020
Human mesenchymal stem cells (hMSCs) were cultured in αMEM with 16.5% exosome-depleted FBS. The MSC conditioned media were collected, and MSC-EVs were precipitated with Total EV Isolation solution. The pellet was resuspended in 10 mM PBS, and the EVs isolated by ultracentrifugation. EVs were also collected from inflammation-educated MSCs (edu-MSC-EVs). Microglia HMC3 cells were challenged with LPS or Aβ oligomers, and the conditioned media of LPS- or AβO-challenged microglia were collected and added to MSCs. Then, the total secretome of proinflammatory-primed hMSCs was collected and the EV fraction isolated.	8-wk-old female hemizygous 5×FAD mice	5×FAD mice were administered PBS, MSC-EVs or edu-MSC-EVs intranasally at a dose of 20 × 10^9^ particles/mouse, once a week for 10 consecutive weeks. Non-carrier (NCAR) mice of the same genetic background were used as control. Mice were sacrificed 1 wk after administration of the last dose (19-wk-old). The brains were analyzed by immunohistochemistry. The Morris water maze test was performed to evaluate mice spatial learning and memory performance. All mice were trained for 5 d (3 trials/day), starting 1 d after the end of treatments.	edu-MSC-EVs treatment enhanced the learning ability of the AD mice. edu-MSC-EV-treatment inhibited microgliosis and astrogliosis in the AD mice. edu-MSC-EVs treatment inhibited the expression of proinflammatory cytokines in the cortex and hippocampus, and inhibited Aβ deposition.	Markoutsa et al., 2021
MSCs were isolated from bone marrow of healthy donors 19–49 years of age. MSCs were expanded in complete culture medium (CCM) of α-MEM with 10% FBS and 1% penicillin/streptomycin. 3D aggregation of MSCs was performed. MSCs at 80%–90% confluence were harvested and seeded in each well of ultra-low attachment 6-well plates. Each well contained 1.0–2.0 × 10^5^ cells with 2 mL CCM with 10% EV-depleted FBS. Cells cultured on a tissue culture plate were used as 2D control. Conditioned medium was collected after 48 h. Enrichment of EVs was performed.	28 nontransgenic (NT) C57BL/6J and 28 transgenic 5×FAD mice, equal numbers of male and female mice, 6 wk of age. After 2 wk of acclimatization, 14 mice of each group received either intranasal sterile saline 5 μL or intranasal EVs (2 × 10^9^ EVs) in 5 μL saline in each nostril every 4^th^ d.	At 4 mon of age, 7 NT + 7 5×FAD saline controls and 7 NT MSC-EV + 7 5×FAD MSC-EV treated mice were tested for memory performance. At 1 d following the completion of these tests, these 28 mice were euthanized, and blood and brains collected. The remaining 28 mice continued intranasal treatments for 2 additional mon. After this, they underwent neurocognitive, behavioral assessment before sacrifice and tissue collections. No toxic or adverse effects were observed in mice treated with MSC-EVs. All mice were euthanized and transcardially perfused first by sterile saline followed by 4% paraformaldehyde. Whole brains were removed.	MSC-EVs ameliorated behavioral deficits in AD mice. MSC-EVs treatment decreased Aβ deposition, and reduced astrogliosis in the brains of AD mice.	Cone et al., 2021
MSCs were isolated from bone marrow of healthy donors, and expanded in culture with low glucose DMEM containing 10% FBS, 2 mM L-glutamine and 1% penicillin/streptomycin. For preconditioning, MSC growth medium was replaced with fresh serum-free (SF)-DMEM for 24 or 48 h with the addition of TNF-α (20 ng/mL) and IFN-γ (25 ng/mL). Untreated MSCs were incubated with SF-DMEM without the addition of proinflammatory cytokines. To isolate EVs (a pool of exosomes and microvesicles), serum-MSC-conditioned medium was collected and subjected to a differential centrifugation procedure.	3×Tg-AD mice, female, 7 mon (*n* = 8, 4/group).	EVs were resuspended in sterile PBS at 300 μg/mL (30 μg corresponding vesicular protein, approximately 15 × 10^9^ vesicles). 3×Tg mice were intranasally administered with EVs or PBS in ~5 μL spurts per nostril. Each mouse received 100 μL of PBS or EVs twice, each injection separated by 18 h (50 μL/dose). After 21 d, each mouse was euthanized, and perfused transcardially with 0.1 M PBS followed by 4% paraformaldehyde.	Based on the assumption that preconditioning represents a key strategy to enhance MSC immunomodulatory functions, cytokine (CYT) preconditioning with 20 ng/mL TNF-α and 25 ng/mL IFN-γ was selected as the MSC stimulation protocol to obtain immunocompetent-derived EVs for *in vivo* experiments. Within 6 h from EV treatment, labelled MSC-EVs were incorporated into microglia and to some extent into neurons in the investigated brain regions. MSC-EVs treatment decreased microgliosis in AD mice, and with a dampening effect on polarization of microglia towards a proinflammatory phenotype.	Losurdo et al., 2020
***Controlled cortical impact***				
EVs were separated by ultracentrifugation from the culture supernatant of mouse microglia BV-2 cells. MiR-711 mimic and mimic NC were transfected into BV-2 cells. The EVs from BV-2 cells were designated as NC-EVs and miR-711-EVs.	C57BL/6 mice, male, 8-wk-old were subjected to a controlled cortical impact (CCI) to induce a repetitive mild traumatic brain injury (rmTBI).	The mice were allocated to different groupings (*n* = 12 mice): sham (without CCI), rmTBI (rmTBI mice injected with equal volume of PBS), NC-EVs (rmTBI mice injected with NC-EVs via a tail vein), miR-711-EVs (rmTBI mice injected with miR-711-EVs via a tail vein). Starting 24 h after the AD model establishment, 40 μg portions of EVs were injected into a tail vein every 3 d. The Morris water maze test was performed 28–32 d after modeling.	miR-711-EVs treatment alleviated cognitive deficits in AD mice. MiR-711-EVs. miR-711-EVs treatment decreased TNF-α expression and increased IL-10 expression, and increased the ratio of microglia M2/M1. miR-711-EVs relieved neurodegenerative changes in AD mice.	Zhang et al., 2020
***In vitro* studies**				
Human ASCs from adipose tissue were cultured in DMEM, 1 g/L D-glucose and GlutaMAX supplemented with 20% FBS. Medium was changed every 3–4 d. For subculture, cells were passaged weekly with 0.05% trypsin/4 mM EDTA. EVs were isolated from the culture medium of ASCs. Mouse bone marrow MSCs were cultured in αMEM medium with GlutaMAX, supplemented with 15% FBS, 1% penicillin-streptomycin and 2 ng/mL recombinant murine basic fibroblast growth factor (rMu bFGF), with changes every 3 d. MSCs culture supernatant containing the EVs was collected every 72 h starting at passage 2 up until passage 8. The EVs were isolated using ultracentrifugation.	Mouse microglia BV-2 cells were seeded at 2.1 × 10^5^ cells/cm^2^ in a 24-well plate and incubated 24 h in DMEM-HG with GlutaMAX and 2% FBS. Microglia cells were primed using 1 μg/mL LPS for 3 h. The cells were washed with culture medium, prior to stimulation with 10 μM Aβ aggregates for 24 h. Unstimulated cells were used as negative control (NC).	Eight human ASC-EVs or mouse MSC-EVs per cell were added to BV-2 cells in the respective wells. As control for the effect of the EVs on BV-2 cells, the cells were incubated with the EVs after priming and with no further stimulation.	MSC-EVs suppressed proinflammatory molecules in BV-2 cells stimulated by Aβ aggregates. MSC-EVs treatment reduced TNF-α and NO levels in Aβ-stimulated BV-2 cells.	Kaniowska et al., 2022
6-mon-old male APP mice (*n* = 4) and age-and gender-matched C57BL/6 wild-type counterparts. When the mice reached 12 mon of age, they were euthanized and sEVs isolated from the hippocampi of the APP and C57BL/6 mice. Cy5-labeled miR-342-5p mimics or mimics NC was transfected into sEVs with Exo-Fect Transfection Kit.	HT22 mouse hippocampal neurons were cultured in DMEM medium with 10% FBS and 2 ng/mL FGF-2 at 37°C with 5% CO_2_.	HT22 cells were incubated with sEVs-APP or sEVs-CTL.	The concentration of miR-342-5p was significantly lower in sEVs-APP compared to that in sEVs-CTL group. sEVs-APP elevated BACE1 mRNA level in HT22 cells more than sEVs-CTL. The protein levels of APP, BACE1 and Aβ42 were higher in cell lysates of HT22 cells treated with sEVs-APP than those treated with sEVs-CTL. HT22 cells that were treated with sEVs-miR-342-5p mimic presented a marked decrease in protein levels of APP, BACE1 and Aβ42 compared with sEVs-APP.	Dong et al., 2022

Aβ: Amyloid-β; AβO: amyloid-β oligomer; AD: Alzheimer’s disease; sAD: sporadic Alzheimer’s disease; ADSC: adipose tissue-derived stem cells; APP: amyloid precursor protein; ASC: adipose stem cells; BACE1: beta-site APP cleaving enzyme 1; BDNF: brain-derived neurotrophic factor; BM-MSC: bone marrow mesenchymal stem cells; CCI: controlled cortical impact; CCM: complete conditioned medium; DG: dentate gyrus; DMEM: Dulbecco’s modified Eagle’s medium; EDTA: ethylene diamine tetraacetic acid; EVs: extracellular vesicles; sEVs: small extracellular vesicles; GABA: gamma-aminobutyric acid; GFAP: glial fibrillary acidic protein; FBS: fetal bovine serum; aFGF: acidic fibroblast growth factor; FGF-2: fibrobast growth factor2; Iba1: ionized calcium-binding adapter molecule 1; i.c.v.: intracerebroventricular; IL-1β: interleukin-1β; IL-6: interleukin-6; IFN-γ: interferon-γ; iPSC: induced pleuripotent stem cells; i.v.: intravenous; LPS: lipopolysaccharide; αMEM: α-minimum essential medium; miR: microRNA; MSC: mesenchymal stem cells; NC: negative control; NCAR: non-carrier; NO: nitric oxide; NT: nontransgenic; PBS: phosphate buffered saline; rmTBI: repetitive mild traumatic brain injury; STZ: streptozotocin; TNF-α: tumor necrosis factor-α.

## Discussion

There are two types of AD, i.e., familial AD and sporadic AD (Ulaganathan and Pitchaimani, 2023). Familial AD often causes symptoms before age 65, while sporadic AD typically affects individuals aged 65 years and older (Reitz et al., 2020). In general, familial AD accounts for only 5% of all cases of AD. Its cause is primarily associated with mutations in specific genes, including APP, presenilin 1 (PSEN1), and presenilin 2 (PSEN2), which are responsible for the formation of amyloid peptides and tau pathology (Akhtar et al., 2024). As the most common AD gene mutation, an abnormal PSEN1 accounts for roughly half of familial AD-causing genes (Lanoiselee et al., 2017; Li et al., 2019). PSEN1 is one of the components of γ-secretase that cleaves APP (Bagaria et al., 2022). By increasing miR-29b-2-5p expression, the PSEN1 gene could be targeted and inhibited, thus reducing the activity of γ-secretase and decreasing amyloid production (Wuli et al., 2022). There is still no effective drug for treating AD. Therefore, it is imperative to develop new therapies that can delay or reverse the disease progression, alleviate the symptoms and improve cognitive function.

Transplantation of neural stem cells, human umbilical cord blood cells, or MSCs improves neuropathology in animal models of AD. Previously, it was shown that neural stem cell transplantation improved the cognitive ability of an Aβ-infused model of AD via neuron differentiation and paracrine action (Cui et al., 2016). MSCs, which can be isolated from various organs, represent the best source of adult stem cells. Transplantation of MSCs into the brain reduced Aβ deposition and restored microglial function in transgenic APP/PSI mice (Kim et al., 2012). Furthermore, MSCs alleviated memory deficits in AD mice by modulating immune responses (Lee et al., 2010). Although stem cells and neural progenitor cells have the potential to generate new cells to replace damaged or lost cells in AD, an alternative proposal is that the paracrine action plays a vital role in the therapeutic effects of stem cell therapy (Phinney and Prockop, 2007). The EVs released from cells are major modulators of intercellular communication in many cell types (EL Andaloussi et al., 2013). Among the EVs, exosomes characterized by a nanosize of 30-150 nm transfer several kinds of functional biomolecules that affect many cellular processes (Montecalvo et al., 2012). Systemic administration of MSC exosomes can improve some neurologic conditions by increasing or lowering the levels of specific miRNAs in neural cells (Xin et al., 2012).

From the PubMed searches, we reviewed two sets of original articles that had EVs or exosomes in their titles.

For the articles on treatment with EVs using these animal models of AD (**Table 1**):

(1) Injection of Aβ_1–42_ oligomer into the brain: MSC-EVs injected into the brain ameliorated cognitive decline, reduced Aβ burden, and decreased neuroinflammation (Sha et al., 2021). Similarly, MSC-EVs-miR-206 antagomir given intranasally alleviated cognitive decline and reduced the Aβ burden (Peng et al., 2024).

(2) Intracerebroventricular injection of STZ: MSC-sEVs injected intracisternally improved memory and cognition, reduced Aβ deposition and neuroinflammation, and decreased neuronal apoptosis (Lin et al., 2024a).

(3) Transgenic animals: APP/PS1 transgenic mice were used in three studies. AEVs given intranasally ameliorated cognitive decline, reduced Aβ burden, and promoted synaptic plasticity (Peng et al., 2022). GABA-treated sEVs administered intravenously ameliorated memory deficits and sEVs-agomir-miR-132 increased neuronal survival (Dou et al., 2021). ADSC-EVs given intranasally alleviated memory and cognitive deficits, reduced Aβ burden, decreased microgliosis, and increased neurogenesis (Ma et al., 2020). 5×FAD mice were used in two studies. edu-MSC-EVs administered intranasally enhanced learning ability, decreased Aβ deposition and inflammation, and inhibited microgliosis and astrogliosis (Markoutsa et al., 2021). MSC-EVs given intranasally ameliorated behavioral deficits, decreased Aβ deposition, and inhibited astrogliosis (Cone et al., 2021). 3×Tg-AD mice were used in one study. Cytokine preconditioned MSC-EVs decreased microgliosis and reduced polarization of microglia to M1 phenotype (Losurdo et al., 2020).

(4) Controlled cortical impact: BV-2 microglia EVs-miR-711 given intravenously alleviated cognitive deficits, decreased inflammation, increased the ratio of microglia M2/M1, and relieved neurodegenerative changes (Zhang et al., 2020).

(5) Addition of Aβ aggregates to microglial cells in culture: MSC-EVs suppressed proinflammatory molecules and reduced NO levels (Kaniowska et al., 2022).

(6) Addition of sEVs from hippocampi of APP mice to hippocampal neurons in culture: sEVs-APP increased protein levels of APP, BACE1 and Aβ in hippocampal neurons. sEVs-APP-agomir-miR-342-5p decreased protein levels of APP, BACE1, and Aβ (Dong et al., 2022).

For the articles on treatment with exosomes using these animal models of AD (**Table 2**):

**Table 2:** Treatment of animal models of AD with exosomes

Isolation of Exo	Animal model of AD	Treatment with Exo	Changes in learning and memory, Aβ burden, inflammation, and neuronal survival in treated animal models	Reference
***In vivo studies***				
***Injection of Aβ_1–42_ oligomer into the brain***				
MSCs were isolated from bone marrow of Wistar rats, 5–6 wk of age. BMSCs were cultured in DMEM with 10% FBS and 1% penicillin/streptomycin at 37°C and 5% CO_2_. The cells were transfected with hsa-miR-29b and grown in the presence of 2.5 mg/mL of antibiotic puromycin. Stable cell lines were cultured in BSA or fresh media that had been depleted of serum exosomes by ultracentrifugation. Serum-free supernatants were collected every 2–3 d and exosomes isolated using ultracentrifugation.	Wistar rats, male, 220–240 g, 6–7 wk, injected with Aβ_1–42_ bilaterally into CA1 region of dorsal hippocampus.	Animals were randomly assigned to four groups (*n* = 6 rats/group): PBS injected (control) group, Aβ-injected group in which 10 μg Aβ was injected bilaterally to generate AD model, exosome-miR-29b group in which Aβ+exosomes derived from the stable BMSCs expressing miR-29b were injected simultaneously, exosome mock in which Aβ+exosomes derived from the stable BMSCs expressing mock vector were injected simultaneously. Aβ_1–42_ was injected bilaterally into the CA1 region of the dorsal hippocampus of anesthetized rats. Injections were carried out over 5 min. At the end of the behavioral tests, the animals were sacrificed and the hippocampal left and right CA1 areas were removed.	The level of miR-29b was significantly increased approximately 2.6-fold in purified exosomes obtained from BMSCs stably expressing miR-29b (exosome-miR-29b) compared to the stable BMSC expressing mock vectors. Cognitive impairment of AD rats was partly recovered by treating with exosome-miR-29b. Transplanting engineered exosomes upregulated miR-29b in hippocampus CA1 region and downregulated the target genes *NAV3* and *BIM*.	Jahangard et al., 2020
***AlCl_3_ given by intragastric intubation***				
Rat bone marrow derived MSCs were cultured in DMEM containing 0.5% human serum albumin but not FBS. Exosomes were obtained from the MSCs conditioned media by ultracentrifugation.	Albino rats, male, 6–9 mon, 250–270 g. AlCl_3_ (17 mg/kg, once daily) in 5 mL distilled water was given by intragastric intubation for 8 wk.	After acclimatizing for 1 wk, 90 rats were randomly divided into six groups. Group 1 (control, n = 35): rats were divided equally into 5 subgroups; subgroup 1a, no intervention; subgroup 1b, intragastric intubation with distilled water 8 wk; subgroup 1c, injected i.p. with 5% PEG-400/5% Tween 80 daily for 4 wk; subgroup 1d, injected i.p. with distilled water daily for 4 wk; subgroup 1e, single i.p. injection of 0.2 mL PBS. Group 2 (AD, n = 15): AD rats were sacrificed 12 wk after AD induction (at end of experiment). Group 3 (AD+rapamycin, n = 10): At 8 wk after AD induction, rats were injected i.p. daily with rapamycin 66 μL/10 g body wt for a final dose of 8 mg/kg. Group 4 (AD+MSC-Exo, n = 10): At 8 wk after AD induction, rats were injected i.p. with 0.5 mL MSC-Exo per rat at a concentration of 100 μg protein/mL. Group 5 (AD+3-methyladenine (3-MA)+ chloroquine, n = 10): At 8 wk after AD induction, rats were injected i.p. with 3-MA (10 mg/kg) and chloroquine (40 mg/kg) once a day for 4 wk. Group 6 (AD+MSC-Exo+3-MA+chloroquine, n = 10): At 8 wk after AD induction, rats were injected i.p. with 3-MA (10 mg/kg) and chloroquine (40 mg/kg) daily until the end of the experiment. Simultaneously, a single dose of MSC-Exo (0.5 mL, 100 μg protein/mL) was injected i.p. During the final wk of the experiment, the novel object test was used to assess memory. At the end of the trial period, rats were anesthetized after fasting for 12 h, perfused with fixative, and the brains removed.	MSC-Exo improved memory in AD rats. The AKT/mTOR signaling pathway is closely associated with autophagy, which is regulated by AMPK and mTOR. MSC-Exo modulated AKT/mTOR signaling pathway in brains of AD rats. MSC-Exo alleviated ultrastructural changes in brain tissue and restored autophagy in AD rats. MSC-Exo suppressed Aβ deposition and tau accumulation in brains of AD rats. MSC-Exo improved neuroinflammation and reduced astrogliosis in AD rats. MSC-Exo improved neurogenesis and histopathology of brains of AD rats. MSC-Exo recovered expression level of miR-126-3p in brain tissue of AD rats.	Ebrahim et al., 2024
***Transgenic animals***				
Mouse BV-2 microglia were treated with 1 μg/mL LPS for 6 h (M0). The M0 was stimulated with 20 ng/mL IL-4 to obtain M2-like microglia and with 100 ng/mL LPS and 10 ng/mL IFN-γ to obtain M1-like microglia. After inducing the phenotypes, the medium of BV-2 cells was replaced with EV-free medium for 48 h. Then, the Exo pellets were collected from the supernatant of M2-like microglia. The Exo from microglia were labeled with PKH67 or DiR for tracking. Exo was isolated from serum of 20 first-visit human AD patients and 20 age-matched healthy controls.	APP/PS1 mice	The Exo isolated from the supernatant of about 1 × 10^7^ cells was used for one injection. Behavioral testing was performed 5–14 d following i.v. Exo treatment.	Exo were localized predominantly in brain and liver. Exo derived from M2-like anti-inflammatory microglia attenuated cognitive impairment and neuroinflammation of AD mice. The levels of miR-223 and YB-1 were increased in the hippocampus of Exo treated group. The level of miR-223 was decreased in both serum and serum-derived Exo of human AD patients.	Wei et al., 2024
Mouse dendritic cells DC2.4 were cultured and, after 72 hours of culture the supernatant was collected, and exosomes extracted. Transduction of dendritic cells with lentivirus containing miR-29b-2 and CD47-CCK or CD47-SST was performed. Supernatants were collected and exosomes extracted.	3×Tg-AD mice, female, 8-mon-old.	Mice were divided into three groups: untreated (UT), DC Exo-treated, and DC miR/CD47-SST-Exo treated. A total of 2.5 × 10^10^ exosomes was injected into the tail vein. At 3 d later, the mice were sacrificed, and the brains removed.	MiR-29b-2 loaded CD47-SST-Exo inhibited the expression of PSEN1 protein and Aβ_1–42_ oligomer protein in the hippocampus and cortex of AD mice. MiR-29b-2/CD47-CCK-Exo inhibited only Aβ_1–42_ oligomer in cortex. The engineered exosomes were shown to have reached the hippocampus.	Lin et al., 2024b
Exosomes isolated from the brain of exercised mice and sedentary mice within 24 h after the last training session.	5×FAD mice, 2-mon-old. C57BL/6J WT male mice were used in all experiments.	Mice were exercised on a treadmill five times a wk for 16 consecutive wk. Mice in the SED groups were left on the treadmill without running for the same duration of time as the EXE groups. Mice were divided into four groups: SED-WT, EXE-WT, SED-5xFAD, EXE-5xFAD. Spatial learning and memory were evaluated by Morris water maze test.	Long-term exercise ameliorated memory impairment and reduced Aβ burden in AD mice. Long-term exercise improved the structure and function of BBB in AD mice. Exosomes from EXE-5×FAD mice improved the function of BBB-associated cells *in vitro*. Five exosomal miRNAs (miR-27-3p, -124-3p, -132-3p, -181-5p, and -532-5p) were selected for RT-qPCR analysis. Only miR-532-5p was found to be differentially expressed in brain-derived exosomes between exercised mice and sedentary mice. AD led to a marked reduction in miR-532-5p expression (SED-5×FAD vs. SED-WT). Long-term exercise increased the level of miR-532-5p in neurons of the cortex and hippocampus of AD mice, but not in astrocytes.	Liang et al., 2023
Mouse microglia BV2 cells were cultured in DMEM/F12 medium with 10% FBS and 1% penicillin/streptomycin at 37°C and 5% CO_2_. For 1070 nm LED 12 mW/cm^2^ treatment, cells received treatment for 7, 14, 21 or 28 min with fluences of 2, 4, 6, or 8 J/cm^2^ at 10 Hz, respectively. Then, cells were maintained in a dark incubator with 5% CO_2_ before harvesting the cells at 4 h for co-culture. 24 h after 1070 nm light treatment, the cell medium of BV2 cells was centrifuged to remove cell debris and the supernatant mixed with ExoQuick TC overnight at 4°C. The mixture was centrifuged repeatedly, and exosome pellets obtained. Mimics or inhibitors of miR-7670-3p were loaded into exosomes using an exosome transfection kit.	5×FAD mice, male, 4 mon old.	Mice were divided into six groups: negative control (WT), sham treatment (5×FAD), 4 J/cm^2^ 1070 nm light irradiation (5×FAD+4 J/cm^2^), 4 J/cm^2^-BV2/mock-Exo treatment (5×FAD+4 J/cm^2^-BV2/mock-Exo), 4 J/cm^2^-BV2/miR-7670-3p mimic-Exo treatment (5×FAD+4 J/cm^2^-BV2/miR-7670-3p mimic-Exo) and 4 J/cm^2^-BV2/miR-7670-3p inhibitor-Exo treatment (5×FAD+4 J/cm^2^-BV2/miR-7670-3p inhibitor-Exo). Mice in the 5×FAD+4 J/cm^2^ group received irradiation of 4 J/cm^2^ at 10 Hz in the 1070 nm light device every second day for 2 mon before behavioral tests. After behavioral tests, mice were euthanized and the brains dissected. Exo were administered intranasally after giving 10 μL per nostril of hyaluronidase (100 U) in PBS to enhance mucous membrane permeability. BV2 Exo 200 μg/mL were suspended in sterile PBS. Approximately 2.8 × 10^9^ exosomes in a total volume of 20 μL were dispensed into the two sides of the nostril in 5 μL spurts separated by 5 min. Intranasal administration was performed every second day and each animal received 26 doses over 2 mon before the behavioral test.	Exposure to 1070 nm light promoted M2 polarization and inhibited M1 polarization in microglia. Treatment with 4 J/cm^2^-BV2/miR-7670-3p mimic-Exo improved memory and cognitive abilities of AD mice. Treatment with 4 J/cm^2^-BV2/miR-7670-3p mimic-Exo attenuated Aβ deposition and neuroinflammation in AD mice. Treatment with 4 J/cm^2^-BV2/miR-7670-3p mimic-Exo increased miR-7670-3p expression in brain tissue. Treatment with 4 J/cm^2^-BV2/miR-7670-3p mimic-Exo protected dendrites and synapses of neurons in AD mice.	Chen et al., 2023
Adipose-derived stem cells (ADSCs) were isolated from adipose tissues of healthy subjects or normal mice. ADSCs were cultured in DMEM with 15% FBS, 100 U/mL penicillin and 100 μg/mL streptomycin at 37°C with 5% CO_2_. Normoxic ADSC cultures were incubated in 95% air (20% O2) and 5% CO_2_. For hypoxia induction, ADSCs were cultured in 94% N_2_, 1% O2, and 5% CO_2_. For ADSC-derived exosome isolation, mouse ADSCs were cultured in FBS-free endothelial cell growth medium (EGM)-2MV supplemented with 1× serum replacement solution for 2 d. The conditioned culture medium was centrifuged and then ultracentrifuged.	APP/PS1 double transgenic mice, B6C3-Tg (APPswe, PSEN1dE9) 85Dbo/J mice, male, 6 wk old.	The 2-mon-old APP/PS1 mice were treated with PBS (AD), exosomes derived from ADSCs (Exo), hypoxia-pretreated ADSCs (HExo), or circ-Epc1-expresing ADSCs (circ-Epc1-Exo) monthly for 2 mon (*n* = 10 mice/group). For all groups, the injection volume was 100 μL. Exosomes from 1 × 10^9^ ADSCs were dissolved in 100 μL PBS. To detect exosome presence in the brain, 3 mice from each group were euthanized at 5 h post-injection to examine brain slices after counterstaining with 4ʹ,6-diamidino-2-phenylindole (DAPI). For exosome tracing, exosomes were labeled with Di1.	Exosomes were found in the hippocampus and cortex at 5 h after injection. HExo treatment improved cognitive function of AD mice. HExo treatment decreased neuroinflammation in brains of AD mice. HExo treatment suppressed neuron apoptosis in hippocampus of AD mice. RT-qPCR showed circ-Epc1 expression was significantly increased in HExo treated AD mice.	Liu et al., 2022
For brain exosome isolation, fresh mouse brains were dissected and treated with papain 20 units/mL in Hibernate E solution (5 mL/brain) for 30 min at 37°C. The brain tissue was gently homogenized in 10 mL cold Hibernate E solution. The brain homogenate was filtered and exosomes in the filtrate collected by ultracentrifugation. Exosomes were isolated from cortical and hippocampal tissues	7-mon-old APP/PS1 mice and control mice (Ctl).		By RT-qPCR, miR-185-5p expression levels were decreased in exosomes derived from AD mouse brains compared with exosomes from Ctl brains. By RT-qPCR, serum miR-185-5p was downregulated in three AD patients compared to three age-/gender-matched controls. RT-qPCR showed expression levels of serum exosomal miR-185-5p were lower in three AD mice than in three age-/gender-matched C57 mice.	Ding et al., 2022
Mouse ADMSCs were cultured in DMEM complete medium at 37°C with 5% CO_2_. MiR-22 mimic was loaded into ADMSCs using transfection reagent. After transfection for 72 h, cells were regularly cultured. ADMSCs were cultured for 72 h to collect conditioned medium, followed by isolation of exosomes by ultracentrifugation.	4-mon-old APP/PS1 double transgenic mice.	APP/PS1 mice were assigned into the Control, Exo and Exo-miR-22 groups (*n* = 10/group). APP/PS1 mice in the Control group were routinely maintained and injected with normal saline. Exosomes used in the Exo group were ADMSC-derived exosomes without transfection with miR-22 mimic. Exosomes used in the Exo-miR-22 group were miR-loaded exosomes after miR-22 mimic transfection. Exo and Exo-miR-22 were injected into the tail vein every 7 d at a dose of 100 μg/mL (50 μL each time), whereas mice in the Control group were injected with 50 μL normal saline each time. Morris water maze test was used to test the memory ability of mice. After 30 d of behavioral examination, mice were euthanized and the brains removed.	Cognitive deficits of AD mice were alleviated by Exo-miR-22. Treatment with Exo-miR-22 decreased inflammatory factors in the peripheral blood and CSF of AD mice. Exo-miR-22 treatment inhibited neuron damage in AD mice.	Zhai et al., 2021
MSCs were obtained from bone marrow of femurs and tibias of C57BL/6 mice, male, 6 wk. MSCs inoculated on the cell culture plate at 5 × 10^5^ cells/60 mm were ischemically preconditioned (PC) for 12 h in a modulator incubator chamber which was flushed with a hypoxic gas mixture 95% N_2_, 5% CO_2_. The medium was collected and centrifuged. The supernatant was mixed with ExoQuick Exosome Precipitation Solution at 4°C overnight.	APP/PS1 double transgenic mice, male, 7-mon-old and WT littermates, male (control).	APP/PS1 mice were treated through the lateral caudate vein by MSC Exo or PC-MSC Exo once every two wk for a total of 8 times (10 mice/grp). The treated dose of exosomes was 150 μg suspended in 80 μL saline. Ten control mice received saline only. To detect the presence of the exosomes in the brain and other organs, APP/PS1 mice, 7-mon-old, were injected with Dil-labeled exosomes from MSC or PC-MSC through the lateral caudate vein. Brain, lungs, and spleen were collected at 5 h after injection. After the treatment was finished, the Morris water maze test was used to assess the spatial memory performance of mice. After the behavior test, mice were anesthetized and perfused with 4% paraformaldehyde, and the brains removed.	In brains collected at 5 h after injection, there was a wide distribution of exosomes throughout the brain, including cerebral frontal cortex and hippocampus. PC-MSC Exo treatment alleviated cognitive and memory impairments in AD mice. PC-MSC Exo treatment alleviated Aβ deposition in cortex and hippocampus of AD mice. PC-MSC Exo treatment increased synaptic protein expression. PC-MSC Exo treatment inhibited microgliosis and astrogliosis. PC-MSC Exo treatment reduced the level of pro-inflammatory cytokines and increased the level of anti-inflammatory cytokines in the brains of AD mice. PC-MSC Exo treatment upregulated the level of miR-21 in brains of AD mice. Overexpression of miR-21 in brains of AD mice rescued memory deficits, decreased Aβ deposition, and downregulated the levels of proinflammatory cytokines.	Cui et al., 2018
***Controlled cortical impact***				
Repetitive head injury was induced for a total of 4 times with a 48-h interval. Sham-operated mice underwent the same procedures except for the impact. Animals were sacrificed at 1, 3, 7, 14, 21, 28, 35, and 42 d post-injury (DPI), cerebral cortex and hippocampus were removed, mixed together, and incubated with 3 mL Hibernate-A cell culture medium. The samples were digested by papain 20 U/mL for 15 min at 37°C, and centrifuged. The supernatants were filtered, Total Exosome Isolation Reagent was added, and incubated overnight at 4°C. Microglial exosomes were harvested by ultracentrifugation. Mouse BV-2 microglia were cultured in DMEM/F12 medium with 10% FBS, 100 U/mL penicillin and 100 mg/mL streptomycin at 37°C. MiR-124-3p mimics were transfected into microglia. The miR-124-3p upregulated exosomes were harvested from the culture medium 48 h later.	C57BL/6 mice, male, 12 wk, 20–25 g. Anesthetized mice were subjected to closed-head injury using the electronic controlled cortical impact device.	Mice were randomly assigned to 4 groups: sham, rmTBI mice, rmTBI mice treated with unedited microglial exosomes (rmTBI+Exo), rmTBI mice treated with miR-124 exosomes (rmTBI+Exo-124). Before treatment, the exosomes were labeled with PKH26. The PKH26-labeled microglial exosomes were then injected i.v. into rmTBI mice via tail vein (3 × 10^10^ in 200 μL PBS per mouse) at 35 DPI. Soluble Aβ1–40 and Aβ_1–42_ levels in extracts of hippocampus were measured at 42 DPI. The novel object test was performed on rmTBI mice at 42 DPI, and the Morris water maze test was performed at 43–47 DPI.	The microglial exosomes harvested from mixed tissue of bilateral cerebral cortex and hippocampus had the characteristic biomarkers for exosomes, including CD9, CD63, and CD81, showing that exosomes were the major component of the isolated EVs from rmTBI mouse brains. MiR-124 level in microglial exosomes from injured brain of mice was altered at different stages after rmTBI. Treatment with microglial exosomes with upregulated miR-124 improved cognitive outcome after rmTBI, and alleviated neurodegeneration in rmTBI mice.	Ge et al., 2020
***In vitro* studies**				
***Treatment with Aβ***				
Mouse cortical neural stem cells (NSCs) were harvested from fetal brain tissue and cultured in NSC proliferation medium. Exosomes were isolated from NSCs using ultracentrifugation.	Human neuroblastoma SH-SY5Y cells were grown to 80% and Aβ1–40 was added to fresh complete culture medium up to a final concentration of 5 μmol/l. Cells were incubated with 5% CO_2_ at 37°C for 24 or 48 h.	1 × 10^5^ SH-SY5Y cells were seeded into the lower chambers of a Transwell coculture system. For the cell treatment, approximately 5 × 10^5^ NSCs were seeded into the upper chambers. The cells were separated by a porous membrane with 0.4 μm pores. In addition, a contact co-culture system for exosomes and SH-SY5Y cells was established. Exosomes were added to the culture medium at 2 μg exosomes per 1 × 10^5^ recipient cells. GW4869 was used to inhibit exosome secretion from NSCs. GW4869 at 20 μM was used to treat NSCs.	After exposure of SH-SY5Y cells to Aβ1–40, the number of cells was reduced, the morphological appearance was changed and cell body shrinkage, rough cell surface, and retracted cell neurites were observed. As the exposure time increased, the morphological changes became more obvious. There was a time-dependent increase in cell apoptosis in the AD model. The apoptosis rate of the AD model cells co-cultured with NSCs or NSC-Exo was reduced. In the *in vitro* AD model, there was a significant decrease in miR-138-5p. When the *in vitro* AD model was co-cultured with NSCs or NSC-Exo, there was increased miR-138-5p in the AD model cells. The effect of NSC-Exo on miR-138-5p expression was almost equivalent to that of NSCs. The downregulation of tau in the NSC-Exo cocultured AD model cells was enhanced when NSCs were pretreated with antagomir miR-138-5p and reversed when pretreated with agomir miR-138-5p. The apoptosis was inhibited with the overexpression of miR-138-5p by agomir but increased with the downregulation by antagomir.	Meng et al., 2023
***Expression of APP mRNA in cells***				
Mouse N2a cells were cultured in 10 cm dish. 24 h before collecting supernatants, the culture medium was replaced with FBS free medium containing DMEM and penicillin 100 U/mL and streptomycin only. Exosomes were isolated from conditioned media by ultracentrifugation.	Full-length APP mRNA was ectopically expressed in N2a cells to mimic the pathological conditions of AD *in vivo*. APP-overexpressed cell-derived exosomes (APP-Exo) or empty vector-transfected (control) cell-derived exosomes (Exo) were collected.		Co-culture of N2a cells with 20 μg/mL APP-Exo for 2 d. increased the expression levels of APP transcripts and APP proteins. RT-qPCR demonstrated a decrease in the level of miR-185-5p in APP-Exo versus Exo.	Ding et al., 2022
***Repetitive scratch injury***				
Mouse BV2 microglia cells were cultured in DMEM/F12 medium containing 10% FBS, 100 U/mL penicillin, and 100 mg/mL streptomycin at 37°C. MiR-124 mimics were transfected into microglia and cultured in DMEM/F12 medium. The miR-124 upregulated Exo were harvested from the culture medium 48 h later.	The repetitive scratch injury model was used to study the impact of microglial exosomes on mouse HT22 hippocampal neurons after rmTBI. Two scratch injuries were conducted with a 12-h interval. The first injury was performed by scratching across the cell surface vertically with a 4 mm space between each line using a pipette tip. The second injury was performed using the same method but scratching horizontally.	The cultured neurons were randomly assigned to four groups: uninjured neurons (control), injured neurons (injury), injured neurons with unedited exosome (I+Exo), injured neurons treated with Exo-124 (I+Exo-124). For exosome treatment, the culture medium of neurons was replaced with the serum-free neurobasal medium after the second scratch injury. Media containing 3 × 10^8^ microglial exosomes were the added to the culture plate.	In the repetitive scratch injury model using cultured HT22 mouse hippocampal neuronal cell line, cell proliferation sharply decreased after the two injuries and gradually recovered to over 90% in 24 h post-injury. The miR-124-3p level in neurons was increased after injury and could be further upregulated by treating with microglial Exo-124 exosomes. The expression levels of APP and Aβ in neurons, and Aβ1–40 and Aβ_1–42_ in the culture medium were increased after injury and were suppressed after treatment with Exo-124 exosomes. Treatment with Exo-124 inhibited neurodegeneration.	Ge et al., 2020

Aβ: Amyloid-β; AD: Alzheimer’s disease; ADMSC: adipose-derived mesenchymal stem cells; ADSC: adipose-derived stem cells; AKT: protein kinase B; AlCl_3_: aluminium chloride; AMPK: AMP-activated protein kinase; APP: amyloid precursor protein; BBB: blood–brain barrier; BMSC: bone marrow stem cells; CCK: cholecystokinin; CSF: cerebral spinal fluid; DC: dendritic cells; DMEM: Dulbecco’s modified Eagle’s medium; DPI: days post-injury; EV: extracellular vesicles; EXE: exercised; Exo: exosomes; F: fasudil; F12: Ham’s F12 nutrient mixture; FBS: fetal bovine serum; GFAP: glial fibrillary acidic protein; HExo: hypoxia-pretreated exosomes; Ibai: ionized calcium binding adapter molecule; IFN-γ: interferon-γ; IL-1β: interleukin-1β, IL-4: interleukin 4; IL-6: interleukin 6; LED: light emitting diode; MSC: mesenchymal stem cells; mTOR: mammalian target of rapamycin; NFkB: nuclear factor kappa-light-chain-enhancer of activated B cells; NS: normal saline; NSC: neural stem cells; PBS: phosphate buffered saline; PC: preconditioned; PEG: polyethylene glycol; rmTBI: repetitive mild traumatic brain injury; RT-qPCR: real time quantitative polymer chain reaction; SED: sedentary; SST: somatostatin; TNF-α: tumor necrosis factor-α; WT: wild type.

(1) Injection of Aβ_1–42_ oligomer into the brain: MSC exosomal (Exo)-miR-29b injected into the hippocampus partly alleviated cognitive impairment and downregulated target genes *NAV3* and *BIM* (Jahangard et al., 2020).

(2) AlCl_3_ given by intragastric intubation: MSC-Exo administered intraperitoneally improved memory, suppressed Aβ deposition and tau accumulation, decreased neuroinflammation and astrogliosis, and improved neurogenesis and histopathology of brains (Ebrahim et al., 2024).

(3) Transgenic animals: There were five studies using APP/PS1 mice. M2-like microglia-Exo injected intravenously attenuated cognitive impairment and neuroinflammation (Wei et al., 2024). Injection of hypoxia-treated ADSCs-Exo improved cognitive function, decreased neuroinflammation, and suppressed neuron apoptosis in the hippocampus (Liu et al., 2022). Expression levels of miR-185-5p were decreased in Exo from AD mouse brains (Ding et al., 2022). ADMSC-Exo-miR-22 injected intravenously alleviated cognitive deficits and inhibited neuron damage (Zhai et al., 2021). Preconditioned ischemically MSC-Exo administered intravenously alleviated memory and cognitive impairments, decreased Aβ deposition, reduced neuroinflammation, inhibited microgliosis and astrogliosis, and increased synaptic protein expression (Cui et al., 2018). There were two studies using 5×FAD mice. Exo from exercised mice improved the function of BBB cells (Liang et al., 2023). Intranasal treatment with 4 J/cm2-BV-2/Exo- miR-7670-3p mimic improved memory and cognitive abilities, attenuated Aβ deposition, decreased neuroinflammation, increased M2/M1 ratio, and protected dendrites and synapses of neurons (Chen et al., 2023). 3×Tg-AD mice were used in one study. Dendritic cells transfected with CD47-cholecystokinin-somatostatin-Exo-miR-29b-2 injected intravenously inhibited the expression of PSEN1 protein and Aβ_1–42_ in the brain (Lin et al., 2024b).

(4) Controlled cortical impact: BV-2 microglia-Exo- miR-124 injected intravenously alleviated cognitive deficit and reduced neurodegeneration (Ge et al., 2020).

(5) Addition of Aβ to cells in culture: NSC-Exo reduced apoptosis and downregulated tau in SH-SY5Y cells (Meng et al., 2023).

(6) Expression of APP mRNA in cells: Co-culture of N2a cells with APP-Exo increased the expression levels of APP transcripts and APP proteins (Ding et al., 2022).

(7) Repetitive scratch injury: Treatment of injured HT22 hippocampal neuronal cells with BV-2 Exo-miR-124 suppressed the expression levels of APP and Aβ in neurons, inhibited neurodegeneration, and suppressed Aβ_1–40_ and Aβ_1–42_ in the culture medium (Ge et al., 2020).

From the above, both the EVs and exosomes preparations used in these studies were effective in the *in vivo* animal models of AD by alleviating memory and cognitive deficits, reducing amyloid deposition and neuroinflammation, and enhancing neuronal survival. A schematic diagram showing how treatment with EVs can improve AD is included in a recent review article (Han et al., 2025). Additionally, the *in vitro* studies showed that EVs and exosomes could reduce inflammation and oxidative stress, lower Aβ_1–40_ and Aβ_1–42_ in the culture medium of the injured neurons, particularly by EVs loaded with miR-342-5p mimic and by exosomes loaded with miR-124, and enhance neuronal survival. RT-PCR analysis of brain tissue in AD models was used in some studies to identify which miRNAs were dysregulated and outcome measures could be enhanced using EVs or exosomes loaded with a specific miR-mimic or antagomir.

It is of interest that in two of the studies, EVs were from MSCs preconditioned by treatment with the proinflammatory cytokines tumor necrosis factor-α and interferon-γ to obtain immunocompetent-derived EVs (Losurdo et al., 2020) and exosomes were from MSCs preconditioned by hypoxia (Liu et al., 2022). The former was shown to induce the microglial M2 polarization state with anti-inflammatory properties, correlating with a neuroprotective profile (Losurdo et al., 2020), while the latter had a greater therapeutic effect on improving cognitive functions by decreasing neuronal damage in the hippocampus more than exosomes from normoxic MSCs, and had altered expressions of circRNAs, including circ-Epc1 (Liu et al., 2022). The switch of macrophages from the M1 to M2 phenotype upon exposure to EVs of human adipose cells exposed to hypoxic conditions had been reported previously (Lo Sicco et al., 2017). In one of the studies, exosomes were obtained from microglial BV2 cells irradiated with different fluences (J/cm^2^) of pulsed near infrared light 1070 nm and loaded with or without mimics/inhibitors of miR-7670-3p. Photo irradiation of microglia at 4 J/cm^2^ 1070 nm for 24 hours promoted M2 polarization and the exosomes increased miR-7670-3p expression in brain tissue (Chen et al., 2023). The top six overexpressed miRNAs in exosomes derived from 4 J/cm^2^ 1070 nm light-treated BV2 cells were miR-9-5p, -185-3p, -7676-3p, -7670-3p, -126a-3p, and -22-5p (Chen et al., 2023). In a study using fasudil treatment of AD mice, miRNA analysis of exosomes in serum of fasudil AD mice revealed 1 upregulated (let-7i-5p) and 7 downregulated miRNAs (miR-130b-3p, novel_63, -19a-3p, -451a, -423-5p, -574-5p, and -466i-5p) (Yan et al., 2024). Fasudil-induced cognitive improvement in AD mice could be due to the manipulation of serum exosomes miRNAs such as miR-19a-3p and miR-451a, and which could be potential targets of fasudil-treatment in AD mice (Yan et al., 2024).

Regarding targeted miRNA therapy, the expression levels of miRNAs in brain tissues of AD mice would reveal those that could be raised by EVs/exosomes loaded with miR-mimic or lowered by EVs/exosomes loaded with miR-antagomir as was performed in some of the studies reviewed.

For instance, in the studies with improved AD outcomes using EVs, miR-206-3p antagomir upregulated various differentially expressed genes that are involved in promoting neuronal and axonal growth, regulating synaptic plasticity, regulating hippocampal memory and inflammatory responses, and upregulating brain-derived neurotrophic factor level (Peng et al., 2024). MiR-132 mimic downregulated cleaved Caspase-3 and Bax expression in neurons and exerted a protective effect, inhibited apoptosis of neurons via interfering with PTEN/AKT/FOXO3 signaling, and regulating Bace1 activity (Dou et al., 2021). MiR-711 mimic decreased Itpkb gene expression and mediated the anti-inflammatory pathway and tau hyperphosphorylation (Zhang et al., 2020). MiR-342-5p targeted *Bace1*, thereby modulating BACE1 expression and ameliorating Aβ formation (Dong et al., 2022). Furthermore, in the studies with improved AD outcomes using exosomes, miR-29b-2 mimic inhibited BACE1 (Lin et al., 2024b). MiR-7670 mimic with 1070 nm light inhibited the expression ratios of p-nuclear factor-κB/nuclear factor-κB and p-p38/p38 proteins and decreased inflammatory cytokines; nuclear factor-κB is an essential inflammatory signal downstream of the mitogen-activated protein kinase signaling pathway (Chen et al., 2023). MiR-22 mimic downregulated the expression of NLR family pyrin domain containing 3 inflammasome key protein (NLR family pyrin domain containing 3 and Caspase 1) (Zhai et al., 2021). MiR-124 mimic targeted the Rela/ApoE signaling pathway in repetitive injured neurons and alleviated neurodegeneration (Ge et al., 2020).

Several important limitations have been identified in the studies reviewed: (1) in some of the studies there were very small sized groups of animals, often with only 6 animals per group; (2) no power calculations were performed to determine what group size is needed to avoid type I and type II errors (Schreffler and Huecker, 2024); (3) where gender was reported, the groups were mainly composed of males; this is significant as, in humans, females are more at risk of developing AD; (4) many of the animal models were based on genetic mutations, which accounts for only 5% of AD cases in humans; a sporadic model of AD was established by giving streptozotocin intracerebroventricularly at a low sub-diabetogenic dose to 11-month-old mice (Kelliny et al., 2023; Lin et al., 2024b).

In summary, the articles reviewed have shown that EVs and exosomes were effective in improving behavior, reducing inflammation and amyloid deposits, and increasing neuron survival in models of AD. The efficacy of treatment could be improved by loading EVs and exosomes with miRNA mimics or antagomirs. Possible use of *in vitro* models would enable various treatments incorporating other miRNA mimics or antagomirs to be evaluated before proceeding to *in vivo* studies. Future studies should be performed with larger animal groups to avoid introducing statistical errors. Additionally, studies with newer models of sporadic AD are needed. Recently, the Thy1-ApoE4/C/EBPβ double transgenic mouse has been found to act as a late-onset sporadic AD model (Qian et al., 2024) and should be used. An ApoE4K1 mouse line given intraperitoneal injections of low-dose lipopolysaccharides for a prolonged period showed a significant decrease in spine density, which marks the early stages of AD and could be used as a late-onset model (Ganesan et al., 2024). Additionally, *in vivo* studies with larger-sized groups of animals and a larger proportion of females to better model human populations would aid the translation of this important research to clinical practice.

A search of the NIH Clinical Trials database (ClinicalTrials.gov) revealed that one study is recruiting for “Saliva and EVs for neurodegenerative diseases” (NCT06869135) and a study of unknown status on “The safety and efficacy evaluation of allogenic adipose MSC-exosomes in patients with Alzheimer’s Disease” (NCT04388982), in which exosomes will be delivered by nasal drip. Regarding treating human patients with AD, intranasal administration of EVs or exosomes may be preferable to intravenous administration due to more direct targeting of the brain and it could become a self-administered treatment (Rossi et al., 2025). Modification of peptides on the surface of EVs and exosomes by binding to receptors on the BBB endothelial cells could increase their brain-targeting properties and the concentration of particles delivered to the brain (Jiang et al., 2024). EVs/exosomes have several advantageous properties, including their biocompatibility and low immunogenicity, ability to cross physiological barriers, and can selectively carry various cargo molecules. Challenges that have to be addressed regarding mass production are upscaling, purity, and quality control (Paolini et al., 2022). Unlike cell-based therapies that are a risk for uncontrolled division and differentiation, EVs/exosomes provide a safer more controllable treatment, and possess greater stability for easier storage. Furthermore, concentrating the therapeutic effect at the target site minimizes off-target side effects on healthy tissues and organs. Single antibody-based therapies cannot deliver multiple therapeutic agents simultaneously or in a combined sequence, and were found to have varying clinical benefit and a high risk of serious side effects (van Dyck, 2018). The BBB severely restricts antibody-based therapies for neurological diseases, preventing antibodies from entering the brain (Zhao et al., 2022). A possible source for isolating EVs/exosomes is human umbilical cord MSCs. They offer significant advantages, having low immunogenicity, high self-renewal rates, and noninvasive collection.

## Data Availability

*Not applicable.*
